# Alterations in skeletal muscle health and biomechanical properties in patients with early rheumatoid arthritis: an exploratory cross-sectional study

**DOI:** 10.3389/fphys.2025.1575689

**Published:** 2025-04-23

**Authors:** Brian J. Andonian, Hailee Patel, Mingzhi Xu, Alyssa M. Sudnick, Johanna L. Johnson, William E. Kraus, George A. Truskey, Kim M. Huffman

**Affiliations:** ^1^ Division of Rheumatology and Immunology, Department of Medicine, Duke University School of Medicine, Durham, NC, United States; ^2^ Duke Molecular Physiology Institute, Duke University School of Medicine, Durham, NC, United States; ^3^ Department of Biomedical Engineering, Duke University, Durham, NC, United States; ^4^ Department of Biomedical Engineering, Texas A&M University, College Station, TX, United States

**Keywords:** rheumatoid arthritis, skeletal muscle, physical function, biomechanical properties, grip strength

## Abstract

**Background:**

Skeletal muscle disease in patients with early-stage rheumatoid arthritis (RA) is understudied. The objective of this study was to identify whether patients with early RA (symptoms <6 months) have impaired skeletal muscle health.

**Methods:**

Participants with early RA (n = 10) and age-, sex-, and BMI-matched healthy controls (n = 10) underwent cross-sectional clinical, physiological, and muscle biomechanical property assessments. Upper and lower extremity muscles underwent *in vivo* passive biomechanical property—tone, stiffness, and elasticity—assessments via myotonometry (MyotonPro®). *In vitro* muscle force production and stiffness were assessed using 3D bioengineered myobundles derived from myoblasts obtained from vastus lateralis muscle biopsies.

**Results:**

Despite similar muscle mass and self-reported physical activity behaviors for patients with early RA and healthy controls, patients with early RA had poorer self-reported physical function, self-reported physical health, and right-hand grip strength (p < 0.05 for all). Early RA muscle tone and stiffness were lower than in controls (p < 0.05) and had an inverse association with prednisone use (rho = −0.72, p = 0.02). While 3D bioengineered myobundle force production and passive stiffness were similar to controls, early RA myobundle stiffness correlated with swollen joint count (rho = −0.67, p = 0.04).

**Conclusion:**

In this exploratory study, patients with early RA exhibited multiple skeletal muscle deficits across clinical, physiologic, and biomechanical domains compared to controls with similar muscle mass and physical activity. *In vivo* and *in vitro* skeletal muscle biomechanical assessments may be useful to identify these deficits to better understand and improve RA muscle health.

## Introduction

Rheumatoid arthritis (RA) is a chronic inflammatory arthritis that is associated with low exercise tolerance, sarcopenia, disability, increased risk of cardiovascular disease, and an overall early aging phenotype (Scott et al., 2010; Pinheiro et al., 2015; Giles et al., 2008). Even when in disease remission, RA patients continue to have low muscle strength and decreased physical function, implicating persistent muscle deficits that define RA skeletal muscle disease (Andonian and Huffman, 2020). The molecular phenotype of RA skeletal muscle disease is characterized by inflammation, altered metabolism, and dysregulated remodeling, which are associated with disease activity, physical inactivity, and disability (Andonian and Huffman, 2020; Huffman et al., 2017; Andonian et al., 2018). Despite advances in understanding the clinical phenotype and molecular underpinnings of RA skeletal muscle disease in patients with long-standing disease, the effects of RA on the skeletal muscle are less well-characterized in pre-clinical and early-stage disease.

Skeletal muscle remodeling, and thus skeletal muscle health, relies on intrinsic muscle biomechanical properties, including passive (i.e., resting) myofascial tone (Masi et al., 2010). The umbrella term myofascial tone includes stiffness—the ability of the tissue to resist deformation in response to an applied force—and elasticity—the tissue’s ability to return to its original form after deformation (Masi et al., 2010). To assess the *in vivo* biomechanical properties of the RA skeletal muscle, we used the MyotonPro® device (https://www.myoton.com). The MyotonPro® is a reliable, valid, handheld, portable, low-cost myotonometer device that delivers a brief, superficial mechanical compression that leads to an impulse oscillation of the underlying muscle. The oscillation is then recorded by the device, which subsequently calculates the tone (oscillation frequency; Hz), stiffness (resistance to the force of deformation; N/m), and other biomechanical properties such as elasticity (inverse of logarithmic decrement characterizing the ability of tissue to return to its initial shape after deformation; relative arbitrary unit) (White et al., 2018; Kelly et al., 2018; Aird et al., 2012; Pruyn et al., 2016). To compare the clinical RA muscle phenotype and *in vivo* biomechanical property findings to an *in vitro* model of the skeletal muscle from patients with early RA, we also evaluated myobundles derived from vastus lateralis muscle biopsy tissue samples which exhibit structural, physiologic, and molecular hallmarks of native skeletal muscle (Madden et al., 2015).

The goals of this study were to investigate 1) the effects of early RA (i.e., less than 6 months from the onset of inflammatory arthritis symptoms) on skeletal muscle health and 2) the potential of both *in vivo* and *in vitro* muscle biomechanical property assessments as tools for monitoring RA skeletal muscle disease. Here, we recruited a unique group of participants with early RA (i.e., less than 6 months from the onset of inflammatory arthritis symptoms) and age-, sex-, and body mass index (BMI)-matched healthy controls for in-depth clinical, physiological, biomechanical property, and myobundle assessments of skeletal muscle health. We hypothesized that the RA skeletal muscle, even in the early stages of the disease, resembles an aged muscle phenotype with increased passive stiffness and decreased elasticity that is associated with decreased muscle mass, decreased muscle strength, and decreased myobundle force production. Early non-invasive identification of this abnormal biomechanical phenotype in RA via myotonometry would then allow for appropriate clinical interventions, including pharmacologic and physical activity prescriptions, which could be better studied in the laboratory via 3D bioengineered myobundle-based experiments.

## Patients and Methods

### Participants

Study participants included adults older than 25 years with early rheumatoid arthritis (n = 10) and healthy age-, sex-, and BMI-matched controls (n = 10). The rheumatoid arthritis participants were all seropositive (rheumatoid factor and/or anti-cyclic citrullinated protein (CCP) antibody positive) and met the 2010 ACR/EULAR Classification Criteria for RA (Aletaha et al., 2010). Participants with early RA had an inflammatory arthritis symptom duration of less than 6 months and had not previously started a biologic disease-modifying antirheumatic drug (bDMARD). Participants with early RA were allowed to take equivalents of prednisone up to 10 mg daily. Early RA criteria were chosen to minimize the effect of medication on skeletal muscle. All participants were not regularly engaging in exercise according to the 2018 US guidelines prior to the start of the study (Medicine ACoS, 2022). Participants using non-aspirin anticoagulants were excluded, given the potential to complicate skeletal muscle biopsy. Participants with uncontrolled thyroid disease, chronic obstructive pulmonary disease (COPD), Parkinson’s disease, congestive heart failure class III and above, and other connective tissue diseases other than from RA (e.g., axial spondyloarthritis and systemic lupus erythematosus) were excluded based on recognized effects on the skeletal muscle.

### Study design

Patients with early RA were recruited directly from outpatient rheumatology clinics at Duke Health in Durham, NC, United States; age-, sex-, and BMI-matched healthy controls were recruited from the community. After obtaining informed consent, participants were enrolled and completed assessments over two study visits, each lasting approximately 3 hours and completed 1 to 2 weeks apart, in a cross-sectional design. This study complied with the Declaration of Helsinki and was approved by the Institutional Review Board of the Duke University Health System.

### Clinical and physiological outcome measures

*Clinical variables and body composition.* Demographics, medications, and comorbidities were assessed via self-report during an initial interview, with confirmation from the computerized medical record. Height was measured via a stadiometer, and weight was measured via a digital scale. Body composition, including fat mass and lean mass, was measured using the BOD POD®. RA disease activity was assessed by an experienced rheumatologist via the disease activity score in 28 joints (DAS-28)—a composite assessment that includes the number of tender and swollen joints on rheumatologist examination, a self-reported assessment of global health (patient global assessment), and erythrocyte sedimentation rate (ESR) (Prevoo et al., 1995). Patient-reported outcomes included the Stanford Brief Activity Survey (SBAS) assessment of on-the-job and leisure-time physical activity behaviors (Taylor-Piliae et al., 2010) and the Patient-Reported Outcome Measurement Information System (PROMIS) assessment of physical function, physical health, mental health, pain intensity, life satisfaction, and fatigue (Bartlett et al., 2015).

*Strength testing*. Strength assessments included dynamometer-assessed grip and quadriceps isometric strength (Cybex HUMAC NORM, Comp Sports Med, Inc., Stoughton, MA). Strength outcomes are reported as average peak force production over three trials.

*Cardiopulmonary exercise testing*. Cardiorespiratory fitness (relative VO_2_ peak) was assessed using maximal treadmill tests by cardiopulmonary gas exchange testing (CPET) and identified as the highest VO_2_ value (mL/kg/min), averaged over 20 s, occurring at a respiratory exchange ratio of ≥1.05.

### *In vivo* skeletal muscle biomechanical property/myotonometry assessments

**FIGURE 1:**
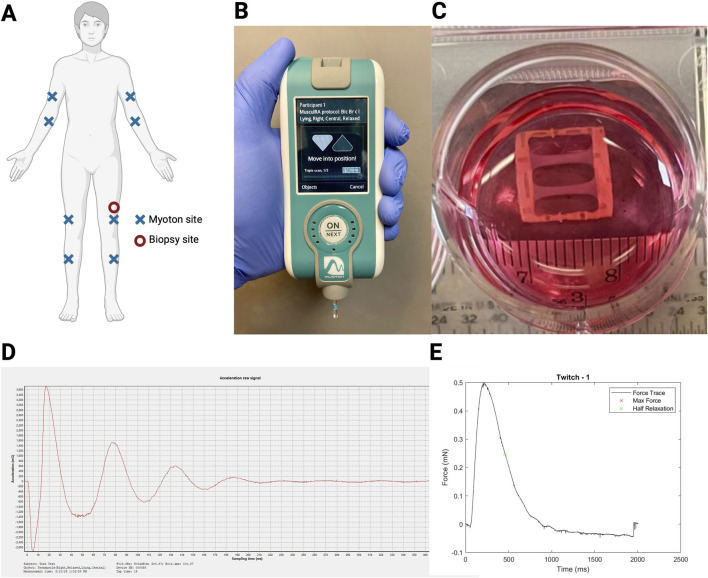
Myotonometry and myobundle experimental setup. **(A)** Experimental setup with myotonometry (Myoton) assessment sites (X; completed in order: right biceps brachii, right forearm flexor, right vastus lateralis, right tibialis anterior, left biceps brachii, left forearm flexor, left vastus lateralis, left tibialis anterior) and biopsy site (circle; left vastus lateralis); **(B)** Myoton assessments completed with participants in the prone position; **(C)** Myobundle culture experimental setup; **(D)** Representative tracing of the Myoton that generated the acceleration signal; **(E)** Representative tracing of the myobundle twitch force measured using a force transducer system.

Participants underwent assessments of passive skeletal muscle tone, stiffness, and elasticity measurements of bilateral biceps brachii, flexor carpi radialis, vastus lateralis, and tibialis anterior using mechanical deformation via myotonometry with the MyotonPro® (Figure 1A). Measurements from multiple tissue sites in the distal and proximal upper and lower extremities were performed to account for variations in subcutaneous fat patterning, given the potential for subcutaneous tissue thickness covering muscle greater than 2 cm to impact MyotonPro® measurements (Frohlich-Zwahlen et al., 2014; Muller et al., 2016). During myotonometry assessments, participants were lying in the supine position and asked to relax as much as possible. At the time of measurement, the MyotonPro® device was held at 90° (i.e., perpendicular to the ground) (Figure 1B) (Andonian et al., 2015). Three measurements were taken for each muscle site assessment, and the mean of the three measurements was recorded. If the coefficient of variation (CV) for repeated measurements was greater than 3.0%, measurements were repeated until a CV of less than 3.0% was achieved. In instances where a CV of less than 3.0% could not be achieved within five assessment trials, the averaged measurement with the lowest CV—always less than 5.0%—was used for analyses. A representative tracing of a Myoton-generated acceleration signal is shown (Figure 1D).

*Principal component analysis*. Given the number of muscles (eight) and the interdependence of the biomechanical properties (tone, stiffness, and elasticity) assessed using myotonometry, principal component analysis (PCA) without rotation was used to reduce the dimensionality of the myotonometry data. Myotonometry (Myoton) factors were created based on PCA on key biomechanical assessment properties with factor loads >0.40. Myoton factors were analyzed and reported if the factor included at least two key biomechanical properties. Myotonometry analyses were primarily reported in terms of factors, given that Myoton values for tone, stiffness, and elasticity across anatomic locations are strongly correlated with each other, and to reduce the impact of spurious results from multiple comparisons. Nonetheless, we performed sensitivity analyses using the subsampling method to create myotonometry factors based on data from 1) control participants only and 2) participants with early RA only.

### Skeletal muscle biopsies

Prior to myotonometry assessments, all participants underwent vastus lateralis muscle biopsies of the left leg using the modified Bergstrom technique (Bergstrom, 1975). Prior to the biopsies, participants completed a washout period of 7 days for those taking anti-platelet agents and 3 days for those taking non-steroidal anti-inflammatory drugs (NSAIDs).

### Myobundle generation

Fresh muscle tissue (up to 200 mg) was minced and treated with 0.05% trypsin-EDTA (Lonza) for satellite cell isolation. Desmin staining was used to assess the purity of the isolation. Myobundles were constructed as follows: Myoblasts (15 million cells/mL) were encapsulated in a fibrin/Matrigel matrix and seeded into nylon frames, which were pinned into polydimethylsiloxane (PDMS) molds treated with 0.2% Pluronic solution and allowed to gel for 30 min at 37°C. After gelling, the myobundles were cultured on a rocker for 4 days in low glucose DMEM (LG-DMEM) supplemented with 8% FBS, SkGM Singlequots (Lonza), and 1.5 mg/mL 6-aminocaproic acid (ACA, Sigma). On day 4, the myobundles were removed from the molds, and the media changed to a Differentiation Medium (DM: LG-DMEM, 2% HS, and 10 μg/mL insulin) supplemented with 2 mg/mL ACA, 100 μM l-carnitine, 50 μM oleate, 50 μM palmitate, 0.28% bovine serum albumin, and 1x antibiotic/antimycotic (Life Technologies). The media was changed every other day. Mature fibers were examined post-shift on day 7.

### Myobundle assessment

Engineered muscle was assessed using a custom-made force measurement setup containing a sensitive optical force transducer and a computer-controlled linear actuator (Thorlabs), as previously described (Figure 1C) (Madden et al., 2015). Samples were stimulated (10 ms, 40 V/cm pulses) to recreate twitch (single pulse; representative tracing shown in Figure 1E) and tetanic (20 Hz for 1 s) contractions and analyzed using a custom MATLAB program. The passive stiffness of the myobundles (mN) was measured using the force transducer system. Fatigue was assessed as the percent of the maximum force 30 s after the application of the tetanus force, normalized to the maximum tetanus force. Myobundle contractile force kinetics were assessed via twitch time-to-maximum force and twitch half relaxation time.

### Statistical analyses

The primary outcome of this exploratory study was the difference in skeletal muscle tone and stiffness in early RA compared to age-, sex-, and BMI-matched control participants. Sample size estimations were based on a previous comparison of muscle biomechanical properties using the MyotonPro®. Compared to younger women, older women had a greater mean passive biceps brachii muscle stiffness (mean ± SD: 302 ± 50 N/m vs. 215 ± 28 N/m, p < 0.001) (22). Assuming a standard deviation of 50 N/m, 10 subjects per group were needed to achieve 80% power (two-sided alpha = 0.05) to detect a clinically meaningful difference in the average muscle stiffness of 49 N/m between the early RA group and their matched controls. Student’s t-tests or Wilcoxon signed-rank tests, depending on data normality, were used to compare RA and control muscle parameters. Spearman’s rho correlations were used to determine relationships between variables. The strength of association for the two groups (RA and controls) was compared using Fisher r-to-z transformations. Complete data (i.e., without any missing data) were analyzed using SAS statistical software (v.9.4). Given the exploratory nature of the present study, p-values less than 0.05 were considered statistically significant without adjustment for multiple comparisons. Effect sizes for comparisons between early RA and healthy control groups were assessed using Hedges’ g statistics, with Hedges’ g > 0.8 considered a large effect size.

## Results

Early RA and healthy control participant demographics and clinical and physiologic outcomes are shown in Table 1. Age, sex, BMI, and race were well-matched between groups. Participants with early RA were rheumatoid factor and/or anti-CCP antibody positive, and average RA disease activity was moderate (i.e., DAS-28 between 3.2 and 5.1). Seven out of 10 participants with early RA were previously treated (i.e., at any time since RA disease onset) and/or being managed at the time of this study with prednisone therapy. At the time of assessment, all participants with early RA were on conventional synthetic (cs) disease-modifying antirheumatic drug (DMARD) therapy, with 9 of 10 on methotrexate and 1 of 10 on sulfasalazine.

**TABLE 1:** Clinical and physiologic outcomes in early RA compared to healthy age-, sex-, and BMI-matched control participants.

Variables	Healthy control (n = 10) Mean (SD)	Early RA (n = 10) Mean (SD)	p-value
Age (years)	53.2 (12.3)	53.6 (12.9)	0.94
BMI (kg/m^2^)	27.7 (4.7)	28.1 (4.2)	0.85
Sex, female n (%)	7 (70%)	7 (70%)	
Ethnicity, n (%) American Indian or Alaska Native Black or African American White	1 (10%) 1 (10%) 8 (80%)	0 (0%) 2 (20%) 8 (80%)	
RF positive, n (%)	N/A	9 (90%)	
Anti-CCP antibody positive, n (%)	N/A	8 (80%)	
RF or anti-CCP positive, n (%)	N/A	10 (100%)	
RA disease duration (months)	N/A	3.7 (1.1)	
RA disease activity (DAS-28)	N/A	4.3 (1.7)	
Prednisone use, n (%)	0/10 (0%)	7/10 (70%)	
csDMARD use, n (%)	0/10 (0%)	10/10 (100%)	
ESR (mm/hour)	**15.3 (13.0)**	**32.4 (19.6)**	0.03*
SBAS-on-the-job physical activity	1.9 (0.6)	1.7 (0.7)	0.48
SBAS-leisure time physical activity	2.6 (0.5)	2.5 (0.7)	0.72
PROMIS-Physical function (T-score)	**55.4 (6.4)**	**39.6 (8.1)**	0.0001*
PROMIS-Physical health (T-score)	**56.2 (6.9)**	**44.6 (8.2)**	0.003*
PROMIS-Mental health (T-score)	54.5 (5.7)	50.2 (9.3)	0.22
PROMIS-Pain intensity (T-score)	**34.2 (4.7)**	**45.7 (8.0)**	0.001*
PROMIS-Life satisfaction (T-score)	57.5 (7.8)	53.7 (9.9)	0.36
PROMIS-Fatigue (T-score)	45.0 (7.4)	49.4 (11.1)	0.31
Lean mass (%)	61.8 (7.7)	60.9 (10.0)	0.84
Hand dominance, right (%)	10 (100%)	8 (80%)	
Grip strength, right hand (kg)	**30.0 (7.1)**	**23.2 (6.8)**	0.04*
Grip strength, left hand (kg)	30.9 (11.8)	25.4 (5.1)	0.20
Isometric knee extension average torque (Nm)	145.2 (48.3)	135.3 (48.1)	0.65
Peak rVO_2_ (ml/kg/min)	27.7 (5.9)	24.3 (6.3)	0.23

Values are shown as mean (SD) unless otherwise noted.

BMI body mass index, CCP cyclic citrullinated peptide, DAS-28 disease activity score in 28 joints, ESR erythrocyte sedimentation rate, PROMIS Patient-Reported Outcomes Measurement Information System, RA rheumatoid arthritis, RF rheumatoid factor, rVO_2_ relative cardiorespiratory fitness, SBAS Stanford Brief Activity Survey.

*P-values *<* 0.05 were considered statistically significant.

Bold values highlight significant results with p < 0.05.

### Clinical outcomes

ESR (mean difference = 17.1 mm/h; 95% confidence interval (CI) = 1.5, 32.7; Hedges’ g effect size = 1.0) was significantly higher in early RA than in healthy control participants (Table 1). While patient-reported on-the-job and leisure-time physical activity were not different between groups, patient-reported physical function (mean difference = −15.8; 95% CI = −22.7, −8.9; Hedges’ g = 2.5) and physical health (mean difference = −11.6; 95% CI = −18.7, −4.5; Hedges’ g = 1.5) were significantly lower and pain intensity (mean difference = 11.5; 95% CI = 5.3, 17.7; Hedges’ g = 1.8) was significantly higher in the early RA group.

### Physiologic outcomes

Right-hand grip strength was significantly lower (mean difference = −6.8 kg; 95% CI = −13.3, −0.2; Hedges’ g = 1.0) in early RA than in healthy control participants (Table 1). Left-hand grip strength (mean difference = −5.5 kg; 95% CI = −14.0, 3.0; Hedges’ g = 0.6), isometric knee extension strength (mean difference = −9.9 Nm; 95% CI = −55.2, 35.4; Hedges’ g = 0.2), and cardiorespiratory fitness (relative peak VO_2_; mean difference = −3.4 mL/kg/min; 95% CI = −9.1, 2.3; Hedges’ g = 0.6) were quantitatively but not significantly lower in early RA. Lean mass was similar between groups (mean difference = −0.9%; 95% CI = −9.3, 7.5; Hedges’ g = 0.1).

### Myotonometry assessments

*In vivo* myotonometry (MyotonPro® device) biomechanical property assessments were reduced via principal component analysis to four factors (Table 2). The four factors constituted 67% of the variance in the myotonometry data and are summarized as Factor 1: upper and lower extremity muscle tone and stiffness; Factor 2: left lower extremity muscle stiffness and elasticity; Factor 3: lower extremity muscle elasticity; and Factor 6: upper extremity muscle elasticity. Sensitivity analysis identified no major differences in factor loadings and grouping of Myoton assessments when including all participants (n = 20) compared to the inclusion of controls (n = 10) or early RA alone (n = 10) (Supplementary Figure S1).

**TABLE 2:** Principal component analysis for myotonometry assessments.

Factor	Myoton assessments within factor	Description	Eigenvalue	Variance	Cumulative variance
1	Right biceps brachii tone, right tibialis anterior stiffness, right tibialis anterior tone, left vastus lateralis tone, right biceps brachii stiffness, left forearm flexor tone, right vastus lateralis tone, right forearm flexor tone, right forearm flexor stiffness, left vastus lateralis stiffness, left forearm flexor stiffness, right vastus lateralis stiffness, left biceps brachii tone, and left biceps brachii stiffness	Upper and lower extremity muscle tone and stiffness	8.29	0.35	0.35
2	Left tibialis anterior stiffness and left vastus lateralis elasticity	Left lower extremity muscle stiffness and elasticity	3.85	0.16	0.51
3	Right vastus lateralis elasticity, left tibialis anterior elasticity, right tibialis anterior elasticity, and left tibialis anterior tone	Lower extremity muscle elasticity	2.58	0.11	0.62
6	Left forearm flexor elasticity and right forearm flexor elasticity	Upper extremity muscle elasticity	1.19	0.05	0.67

Myotonometry assessments are ordered from highest to lowest absolute factor load.

Bold values highlight significant results with p < 0.05.

**FIGURE 2:**
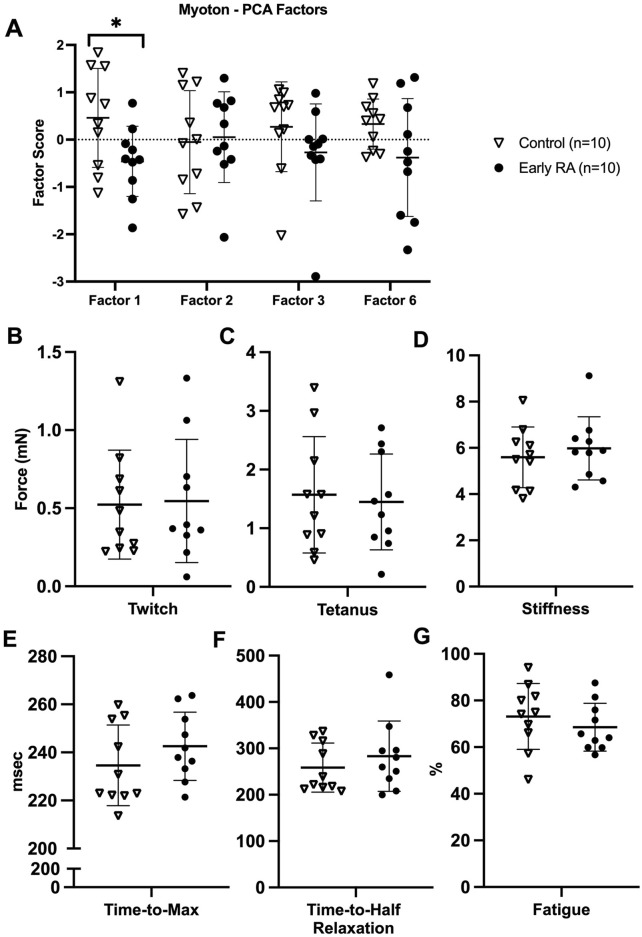
Myotonometry and myobundle outcomes in early rheumatoid arthritis compared to healthy age-, sex-, and BMI-matched control participants. **(A)** Myotonometry (Myoton) factors in early rheumatoid arthritis (RA) *versus* healthy control participants (Myoton-Factor 1 upper and lower extremity muscle tone and stiffness, Myoton-Factor 2 left lower extremity muscle stiffness and elasticity, Myoton-Factor 3 lower extremity muscle elasticity, Myoton-Factor 6 upper extremity muscle elasticity); **(B–G)** myobundle assessments in early rheumatoid arthritis *versus* healthy control participants. All graphs are presented as individual participant values and brackets with mean ± standard deviation. *p < 0.05.

Myoton-Factor 1 (muscle tone and stiffness) was significantly less in early RA compared to healthy control participants (mean difference = −0.92; 95% CI = −1.77, −0.07; Hedges’ g = 1.0) (Figure 2A) (Supplementary Table S1). Other myotonometry factors (Myoton-Factors 2, 3, 6) were similar between groups.

**FIGURE 3:**
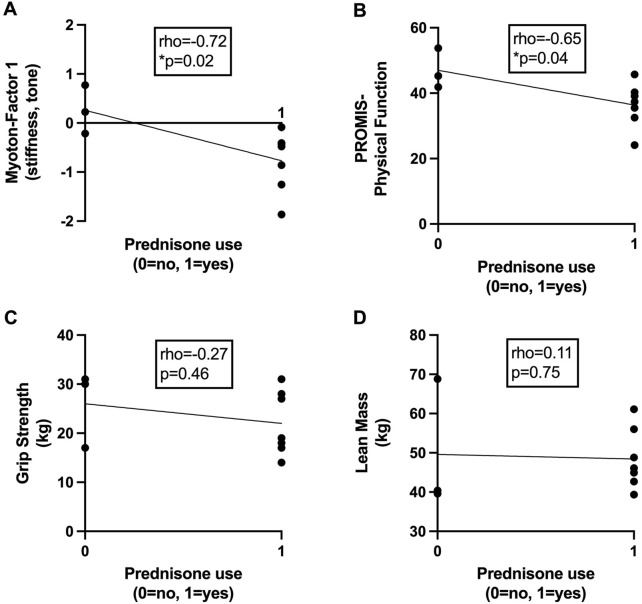
Associations between prednisone use and markers of skeletal muscle health in early rheumatoid arthritis. Scatter plots depict relationships between current or previous use of prednisone (x-axis) with **(A)** skeletal muscle tone and stiffness assessed via myotonometry (Myoton-Factor 1), **(B)** self-reported physical function (PROMIS), **(C)** right-hand grip strength (kg), and **(D)** lean mass (kg) (y-axis) in participants with early rheumatoid arthritis (RA) (n = 10). Associations were calculated using Spearman’s rho correlation coefficients. *p < 0.05.

Among participants with early RA, low muscle tone and stiffness (Myoton-Factor 1) were associated with previous or current prednisone use of up to 10 mg daily (Spearman rho = −0.72, p = 0.02; Figure 3A). Prednisone use was also associated with poor patient-reported physical function (rho = −0.65, p = 0.04; Figure 3B) but not handgrip strength (rho = −0.27, p = 0.46; Figure 3C) or lean mass (rho = 0.11, p = 0.75; Figure 3D). Early RA lower extremity muscle elasticity (Myoton-Factor 3) was associated with longer RA disease duration (rho = 0.64, p = 0.04) (Table 3).

**TABLE 3:** Correlations between myotonometric muscle biomechanical properties and clinical outcomes in patients with early RA (n = 10).

Variables	Myoton-Factor 1	Myoton-Factor 2	Myoton-Factor 3	Myoton-Factor 6
Age (years)	−0.22	−0.18	−0.26	−0.08
BMI (kg/m^2^)	−0.56	0.59	−0.05	−0.06
Male sex	−0.04	0.34	0.11	0.11
RA disease duration (months)	−0.50	0.12	0.64*	0.07
RA disease activity (DAS-28)	−0.27	0.01	0.58	0.47
Tender joints	−0.35	−0.16	0.60	0.62
Swollen joints	−0.03	0.04	0.36	0.49
Patient global assessment	−0.01	0.36	0.37	0.02
ESR (mm/hour)	−0.13	0.18	−0.35	0.03
Prednisone use, n (%)	−0.72*	−0.11	−0.11	0.49
SBAS-on-the-job physical activity	0.22	0.36	−0.34^#^	−0.08
SBAS-leisure time physical activity	0.38	0.13	−0.19	−0.09
PROMIS-Physical function (T-score)	0.36	−0.03	−0.01	−0.30
PROMIS-Physical health (T-score)	0.27	−0.40	0.11	0.05
PROMIS-Mental health (T-score)	0.28^#^	−0.38	−0.15	0.13
PROMIS-Fatigue (T-score)	−0.30	0.07	−0.21	0.14
PROMIS-Pain intensity (T-score)	−0.46^#^	−0.23	0.34	0.04
PROMIS-Life satisfaction (T-score)	−0.41	−0.26	−0.05	0.44
Lean mass (%)	0.37	−0.05	0.02	0.2
Grip strength, right hand (kg)	0.35	0.29	−0.13	−0.31
Grip strength, left hand (kg)	0.24	0.19	−0.18	−0.15
Isometric knee extension average torque (Nm)	0.49	0.19	0.05	−0.30
Peak rVO_2_ (ml/kg/min)	0.55	0.02	0.31	0.09

Values are shown as Spearman’s rho correlations.

BMI body mass index, DAS-28 disease activity score in 28 joints, ESR erythrocyte sedimentation rate, Myoton-Factor 1 upper and lower extremity muscle tone and stiffness, Myoton-Factor 2 left lower extremity muscle stiffness and elasticity, Myoton-Factor 3 lower extremity muscle elasticity, Myoton-Factor 6 upper extremity muscle elasticity, PROMIS Patient-Reported Outcomes Measurement Information System, RA rheumatoid arthritis, rVO_2_ relative cardiorespiratory fitness, SBAS Stanford Brief Activity Survey.

*P-value < 0.05 for correlations in patients with early RA (n = 10) using Spearman’s rho.

^#^P-value < 0.05 for differences in the strength of association for two groups (early RA versus healthy controls) compared using Fisher r-to-z transformations.

Bold values highlight significant results with p < 0.05.

Among healthy control participants, muscle tone and stiffness (Myoton-Factor 1) were associated with poorer patient-reported mental health (rho = −0.6, p = 0.04) and higher pain intensity (rho = 0.83, p = 0.003); lower extremity muscle elasticity (Myoton-Factor 3) was associated with higher on-the-job physical activity (rho = 0.75, p = 0.01); the strength of association differed significantly between early RA and healthy control groups (Fisher r-to-z transformation p < 0.05 for all) (Table 3).

### Myobundle assessments

Overall, myobundle assessments did not differ between early RA and healthy control participants, including for myobundle twitch force (Figure 2B), tetanus force (Figure 2C), stiffness (Figure 2D), time-to-maximum force (Figure 2E), time-to-half relaxation (Figure 2F), and fatigue (Figure 2G).

Among participants with early RA, myobundle twitch and tetanus force were associated with male sex (rho = 0.65, p = 0.04; rho = 0.72, p = 0.02, respectively); the strength of association between twitch force and male sex differed significantly in early RA compared to the healthy control group, with twitch force only associated with male sex in RA (Fisher r-to-z transformation p = 0.03) (Table 4). Lower myobundle stiffness and time-to-maximum force were associated with higher RA swollen joint counts (rho = −0.67, p = 0.04; rho = −0.67, p = 0.02, respectively). Low early RA myobundle stiffness were also associated with low right-hand grip strength (rho = 0.68, p = 0.03).

**TABLE 4:** Correlations between myobundle assessments with clinical outcomes in patients with early RA (n = 10).

Variables	Twitch (mN)	Tetanus (mN)	Stiffness (mN)	Time-to-max (msec)	Time-to-half relaxation (msec)	Fatigue (%)	
Age (years)	−0.13	−0.20	0.24	0.33	−0.18	−0.21	
BMI (kg/m^2^)	0.25	0.21	−0.35	−0.15	−0.47	−0.03	
Male sex	0.65* ^**#**^	0.72*	0.34	0.04	−0.34	−0.49	
RA disease duration (months)	−0.19	−0.23	−0.29	0.07	−0.09	−0.01	
RA disease activity (DAS-28)	0.19	0.32	−0.33	−0.53	−0.05	0.09	
Tender joints	0.10	0.23	−0.11	−0.61	−0.14	0.22	
Swollen joints	0.14	0.22	−0.67*	−0.66*	−0.02	0.36	
Patient global assessment	0.36	0.47	−0.15	−0.19	−0.02	−0.24	
ESR (mm/hour)	−0.13	0.01	−0.20	−0.25	−0.18^#^	−0.08	
Prednisone use, n (%)	−0.19	−0.04	−0.04	−0.42	−0.19	−0.19	
SBAS-on-the-job physical activity	0.10	0.16	−0.14	−0.18	0.04	0.06	
SBAS-leisure time physical activity	−0.31	−0.31	0.15	−0.32	−0.60	0.22	
PROMIS-Physical function (T-score)	−0.14^#^	−0.28^#^	−0.31	0.05	0.22	0.07	
PROMIS-Physical health (T-score)	−0.46	−0.58	−0.28	−0.14	0.10	0.57	
PROMIS-Mental health (T-score)	−0.43	−0.51	−0.17	−0.16	−0.02	0.52	
PROMIS-Fatigue (T-score)	0.50	0.58	0.32	0.29	0.24	−0.50	
PROMIS-Pain intensity (T-score)	0.17	0.30	0.06	−0.13	−0.01	−0.29^#^	
PROMIS-Life satisfaction (T-score)	−0.08	−0.08	−0.48	−0.14	0.04	0.33	
Lean mass (%)	0.22	0.35	0.30	−0.10	0.10	−0.12	
Grip strength, right hand (kg)	0.27	0.27	0.68*	0.26	−0.39	−0.48^#^	
Grip strength, left hand (kg)	0.13	0.21	0.42	−0.05	−0.09	−0.42^#^	
Isometric knee extension average torque (Nm)	−0.20	−0.09	0.28	−0.18	−0.18	−0.20^#^	
Peak rVO_2_ (ml/kg/min)	0.19	0.27	0.22	−0.21	0.07	0.03^#^	

Values are shown as Spearman’s rho correlation coefficients.

BMI body mass index, DAS-28 disease activity score in 28 joints, ESR erythrocyte sedimentation rate, PROMIS Patient-Reported Outcomes Measurement Information System, RA rheumatoid arthritis, rVO_2_ relative cardiorespiratory fitness, SBAS Stanford Brief Activity Survey.

*P-value < 0.05 for correlations in patients with early RA (n = 10) using Spearman’s rho.

^#^P-value < 0.05 for differences in the strength of association for two groups (early RA versus healthy controls) compared using Fisher r-to-z transformations.

Bold values highlight significant results with p < 0.05.

Among healthy control participants, myobundle twitch and tetanus force was associated with higher self-reported physical function (rho = 0.75, p = 0.01; rho = 0.77, p = 0.009, respectively); strength of association was significantly different between groups (Fisher r-to-z transformation p = 0.04 and p = 0.01, respectively) (Table 4). Healthy control myobundle fatigue was associated with lower pain intensity, grip strength, isometric knee extension strength, and cardiorespiratory fitness (rho>0.67, p < 0.03 for all); the strength of association was significantly different between groups (Fisher r-to-z transformation p < 0.05 for all).

Early RA *in vitro* myobundle assessments were generally not associated with *in vivo* myotonometric muscle biomechanical property assessments (Supplementary Table S2). As an exception, lower myobundle time-to-half relaxation was associated with left lower extremity muscle stiffness and elasticity (Myoton-Factor 2) (rho = −0.64, p = 0.04).

## Discussion

In an exploratory cross-sectional study design, patients with early RA (i.e., less than 6 months from inflammatory arthritis symptom onset prior to starting bDMARD therapy) were compared to a well-phenotyped group of healthy controls indistinguishable by age, sex, and BMI. The early RA group reported poorer self-reported physical function and physical health. Participants with early RA also showed trends toward lower objective muscle strength and cardiorespiratory fitness despite similar muscle mass and physical activity behaviors between groups. Furthermore, a composite assessment of early RA myotonometry-based biomechanical properties identified reduced skeletal muscle tone and stiffness compared to healthy controls. Taken together, participants with early RA exhibited skeletal muscle disease characterized by alterations in multiple aspects of muscle health, including physical function, muscle strength, muscle tone, and stiffness.

Early RA skeletal muscle disease appears to be a distinct clinical problem separate from aging alone. Muscle in both longstanding RA and aging is hallmarked by loss of mass, decreased strength, and poor physical performance (Andonian and Huffman, 2020). We expected early RA to exhibit this prematurely aged muscle phenotype in association with reduced elasticity and increased tone and stiffness (Agyapong-Badu et al., 2016; Eby et al., 2015). We theorized that the increased passive stiffness of early RA skeletal muscle could have several direct effects consistent with early aging. For example, muscle cells divide and differentiate at specific values of extracellular matrix stiffness (Lin et al., 2016). In aging, increases in muscle stiffness lead to satellite cell activity decline and subsequently decreased muscle repair (Lacraz et al., 2015). However, compared to controls, the early RA group had less muscle tone and stiffness, along with poorer strength, self-reported physical function, and self-reported physical health. These early RA muscle deficits were present despite participants with early RA and healthy controls having similar physical activity behaviors and muscle mass. Thus, our findings suggest that 1) the pathophysiology of skeletal muscle deficits may differ between early-stage RA, established RA, and aging, and 2) early RA muscle health may require unique assessment, monitoring, and therapeutic strategies.

Myotonometry is a novel clinical tool for the assessment of skeletal muscle health in RA. As assessed by myotonometry, passive muscle stiffness is altered in multiple other pathological conditions, including Parkinson’s disease, stroke, and ankylosing spondylitis, but had not yet been previously evaluated in RA (Andonian et al., 2015). Here, in the first analysis of myotonometry-based biomechanical properties in RA, patients with early RA exhibited low muscle tone and stiffness. In exploratory analyses, altered RA muscle biomechanical properties were associated with current or previous prednisone use. Although both acute and chronic use of corticosteroids (e.g., prednisone) are recognized causes of myopathy in RA (Hanaoka et al., 2012; Lemmey et al., 2018), prednisone use was not highly correlated with muscle mass or strength in this early RA cohort (data not shown). Thus, myotonometry may serve as an important tool to screen, evaluate, and monitor corticosteroid-induced myopathy. Additionally, early RA disease duration was associated with myotonometry-determined muscle elasticity; this finding suggests that, even early in the RA disease course, myotonometry informs clinicians of the impact of RA on skeletal muscle. Myotonometry assessments of the skeletal muscle are relatively quick (i.e., can be performed in less than 5 min), simple to perform (i.e., only require an examination table; use of the device requires minimal training), and are relatively inexpensive. Thus, implementing myotonometry in the clinic is feasible to include in the routine clinical management and monitoring of RA, especially as it impacts skeletal muscle health.

In contrast to the early RA group, in healthy controls, greater muscle tone and stiffness were associated with poorer mental health and higher pain scores, while greater muscle elasticity was linked to more self-reported physical activity. These associations in health controls are in accordance with previous findings of skeletal muscle deficits with aging and the “aged muscle phenotype.” These associations are also consistent with patients with mental health disorders and fibromyalgia, who commonly report muscle tension and stiffness (Carneiro et al., 2017; Kendall et al., 2002). Interestingly, none of the participants with early RA in this study had concomitant fibromyalgia. Thus, RA has a unique myotonometry profile that may offer an opportunity for comprehensive longitudinal assessment to isolate the muscle effects of RA inflammatory disease from those of comorbid fibromyalgia and mental health disorders.

In contrast to differences in myotonometry, we observed few early RA *versus* healthy control differences in myobundle assessments. In a previous study, myobundles from patients with long-standing RA exhibited greater force production and enhanced cytokine production than controls (Oliver et al., 2022). As myobundles are generated from isolated myoblasts, early RA muscle clinical and physiologic muscle deficits may result from impacts on non-myocyte components of muscle tissue such as collagen, fibroblasts, adipocytes, fibro-adipogenic progenitors, and resident immune cells. However, myobundle stiffness and time-to-maximum force were associated with RA-swollen joints at the time of muscle biopsy, indicating that the inflammatory burden of RA may impact myoblast function and subsequent myobundle formation both acutely and over time. Additionally, in healthy controls, but not early RA, myobundle twitch and tetanus force and fatigue were linked with better muscle performance (i.e., self-reported physical function, muscle strength, and cardiorespiratory fitness); these differential associations of myobundle assessments between groups highlight potentially unique impacts of RA on the skeletal muscle that are imparted to muscle myoblasts and throughout myogenesis.

The 3D bioengineered myobundle system is a novel assessment tool for the long-term effects of RA on skeletal muscle, including those related to various therapeutic modalities and hormones. For example, when myobundles derived from a healthy (i.e., non-RA) cohort were exposed to the medication chloroquine, an anti-malarial drug that is also used as an anti-inflammatory therapy for RA, there was a reduction in myobundle contractile force (Madden et al., 2015). A future similar study of myobundles with tissue derived from patients with RA holds promise to better characterize the effects of RA therapies (e.g., corticosteroids, DMARDs, exercise) on the RA skeletal muscle. In the present study, myobundle twitch force was also associated with male sex in early RA but not in the healthy control group, suggesting potentially important sex and hormonal factors contributing to RA muscle disease that can be further evaluated via the myobundle platform. With further study, the myobundle system has the potential to directly inform how RA pharmacotherapies and combinations thereof impact skeletal muscle (e.g., changes in myobundle force production with tumor necrosis factor-alpha inhibitor *versus* Janus kinase inhibitor therapy *versus* combinations). A better understanding of the impact of RA medications on skeletal muscle health, along with the incorporation of RA muscle assessments (e.g., myotonometry) into clinical practice, should improve the personalized clinical care of patients with RA.

This study reports a novel assessment of skeletal muscle health across clinical and physiological domains; however, multiple limitations need to be considered. First, the small sample size of participants with early RA and matched healthy controls was necessary for the degree of in-depth phenotyping presented but limits the generalizability of the findings. Second, although participants with early RA were excluded if they had already been prescribed biologic DMARD therapy, many were currently or previously on prednisone therapy, and all were on csDMARD therapy at the time of assessment, thus limiting analyses of the effect of RA itself (i.e., without RA medication) on the skeletal muscle. Third, patients with long-standing RA were not included; future studies will aim to assess the longitudinal impact on muscle biomechanical properties and myobundle assessments in RA skeletal muscle disease. Finally, the findings presented in the current study were not linked to skeletal muscle molecular assessments; future combined analyses with muscle multi-omic assessments hold promise to better understand the specific muscle molecular-to-physiologic pathways impacted by RA (Huffman et al., 2017).

## Conclusion

In this exploratory study, patients with early RA exhibited deficits in skeletal muscle health across multiple domains, including clinical, physiological, and muscle biomechanical properties. Assessments of bioengineered 3D myobundles did not differ between early RA and age-, sex-, and BMI-matched healthy control groups and were not highly associated with muscle biomechanical properties assessed *in vivo* via myotonometry. However, differential RA–control group associations of myobundle force production with inflammation and physical performance suggest unique features of RA skeletal muscle disease that are imparted to myoblasts and subsequent myobundle generation. Further comprehensive translational studies of early recognition, monitoring, and intervention for RA skeletal muscle disease are needed to improve and personalize care for patients with RA.

## Data Availability

The raw data supporting the conclusions of this article will be made available by the authors without undue reservation.
