# Supplementary material for: Alterations in skeletal muscle health and biomechanical properties in patients with early rheumatoid arthritis: an exploratory cross-sectional study
Source: Front Physiol. 2025 Apr 23;16:1575689. doi: 10.3389/fphys.2025.1575689 (PMC12055543; doi:10.3389/fphys.2025.1575689)

Supplementary Material

| **Supplemental Table 1**: Myotonometry outcomes in early RA compared to age-, BMI-, sex-matched healthy control participants | | | | |
| --- | --- | --- | --- | --- |
| Variables |  | Healthy Control (n=10) | Early RA (n=10) | p-value |
| Tone (Hz) |  |  |  |  |
| Right biceps brachii |  | 15.6 (1.8) | 14.3 (1.3) | 0.07 |
| Right forearm flexor |  | 18.5 (1.7) | 17.0 (2.3) | 0.12 |
| Right vastus lateralis |  | 19.0 (3.6) | 16.6 (2.5) | 0.10 |
| Right tibialis anterior |  | 27.9 (4.7) | 25.1 (4.7) | 0.20 |
| Left biceps brachii |  | 17.0 (1.5) | 15.9 (1.4) | 0.12 |
| Left forearm flexor |  | 19.9 (3.1) | 18.3 (2.6) | 0.22 |
| Left vastus lateralis |  | **22.3 (4.1)** | **18.8 (2.0)** | **0.03*** |
| Left tibialis anterior |  | 26.4 (4.0) | 27.8 (2.4) | 0.35 |
| Stiffness (N/m) |  |  |  |  |
| Right biceps brachii |  | **275.3 (34.9)** | **232.8 (16.1)** | **0.004*** |
| Right forearm flexor |  | 355.4 (42.1) | 322.3 (75.3) | 0.24 |
| Right vastus lateralis |  | 359.6 (66.2) | 312.3 (48.2) | 0.08 |
| Right tibialis anterior |  | 576.7 (115.1) | 516.3 (102.7) | 0.23 |
| Left biceps brachii |  | 296.1 (34.3) | 270.7 (31.3) | 0.10 |
| Left forearm flexor |  | 382.2 (77.6) | 347.3 (74.3) | 0.32 |
| Left vastus lateralis |  | **402.9 (111.0)** | **317.2 (48.4)** | **0.04*** |
| Left tibialis anterior |  | 566.5 (103.1) | 556.4 (69.3) | 0.80 |
| Decrement (inverse of elasticity; relative unit) |  |  |  |  |
| Right biceps brachii |  | 1.29 (0.30) | 1.27 (0.29) | 0.89 |
| Right forearm flexor |  | 1.21 (0.20) | 1.31 (0.30) | 0.41 |
| Right vastus lateralis |  | 1.61 (0.23) | 1.80 (0.32) | 0.15 |
| Right tibialis anterior |  | 1.70 (0.28) | 1.68 (0.30) | 0.90 |
| Left biceps brachii |  | 1.45 (0.41) | 1.30 (0.14) | 0.28 |
| Left forearm flexor |  | 1.39 (0.18) | 1.37 (0.21) | 0.84 |
| Left vastus lateralis |  | 1.46 (0.23) | 1.49 (0.37) | 0.82 |
| Left tibialis anterior |  | 1.44 (0.48) | 1.74 (0.46) | 0.18 |
| *Values are shown as mean (SD). Note: decrement is the inverse of elasticity. RA rheumatoid arthritis, BMI body mass index, Hz natural oscillation frequency, N/m dynamic stiffness*  ********p-values < 0.05 were considered statistically significant* | | | | |

| **Supplemental Table 2**: Correlations between muscle myotonometry factors with myobundle assessments in patients with early rheumatoid arthritis (n=10) | | | | | | |
| --- | --- | --- | --- | --- | --- | --- |
| Variables | Twitch  (mN) | Tetanus  (mN) | Stiffness  (mN) | Time-to-max (msec) | Time-to-half relaxation  (msec) | Fatigue  (%) |
| Myoton-Factor 1 | -0.14 | -0.22 | 0.36 | 0.16 | 0.16 | 0.19 |
| Myoton-Factor 2 | 0.28 | 0.31 | -0.37 | -0.12 | **-0.64*** | -0.26 |
| Myoton-Factor 3 | -0.31 | -0.28 | 0.08 | -0.27 | 0.22 | 0.13 |
| Myoton-Factor 6 | 0.36 | 0.43 | -0.14 | -0.45 | 0.03 | 0.45 |

*Values are shown as Spearman’s rho correlation coefficients. Factor 1 components (in order of highest to lowest factor load): right biceps brachii tone, right tibialis anterior stiffness, right tibialis anterior tone, left vastus lateralis tone, right biceps brachii stiffness, left forearm flexor tone, right vastus lateralis tone, right forearm flexor tone, right forearm flexor stiffness, left vastus lateralis stiffness, left forearm flexor stiffness, right vastus lateralis stiffness, left biceps brachii tone, left biceps brachii stiffness; Factor 2 components: left tibialis anterior stiffness, left vastus lateralis elasticity; Factor 3 components: right vastus lateralis elasticity, left tibialis anterior elasticity, right tibialis anterior elasticity, left tibialis anterior tone; Factor 6 components: left forearm flexor elasticity, right forearm flexor elasticity*

**p-value < 0.05 for correlations in patients with early RA (n=10) using Spearman’s rho*

*^#^p-value < 0.05 for differences in the strength of associations for two groups (early RA versus healthy controls) compared using Fisher r-to-z transformations*

**Supplemental Figure 1: Representative visualization of principal components analysis of myotonometry factors 1 and 2.** Plots depict factor load values from principal components analysis (PCA) for myotonometry assessments included in Factor 1 (x-axis) compared to those included in Factor 2 (y-axis) in (A) the total study population (n=20), (B) control participants only (n=10), and (C) participants with early rheumatoid arthritis (RA) (n=10). Individual myotonometry assessments with factor load values greater than 0.40 were included in final Factor selection.


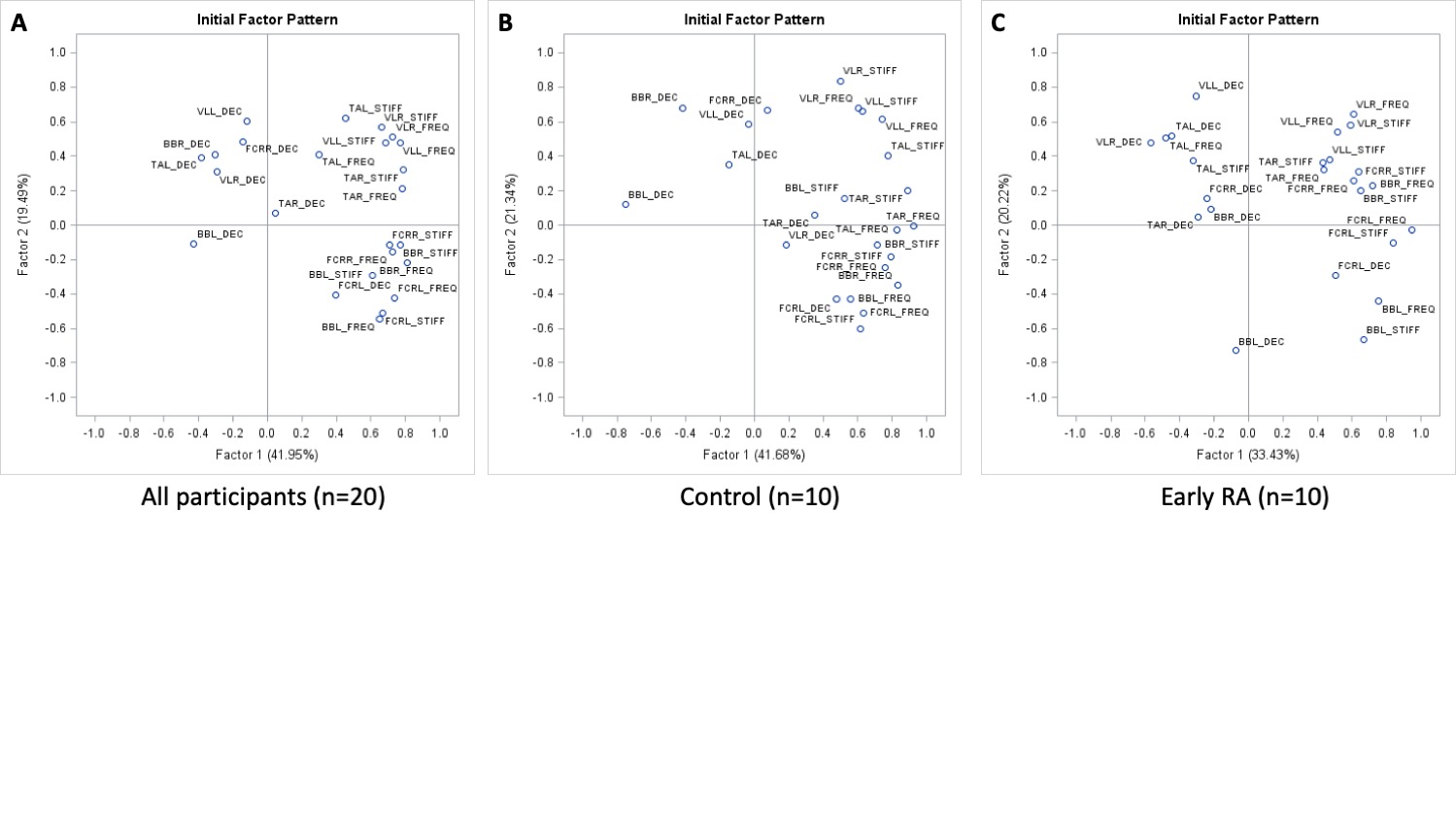

Supplement: Supplementary file 1 [file DataSheet1.docx]
